# Gluttony

**Published:** 1858-04

**Authors:** 


### GLUTTONY.

We are told of some Roman ruler, that he loved eating so well, that he often took an emetic soon after a dinner, in order to have the pleasure of a second repast. Some modern animals manage the thing rather differently, they eat a considerable time after they are full; then, like a gorged Esquimaux or anaconda, lie down in utter stupor, and sleep it off. This, perhaps, proves, that when a man is full he may make himself fuller: such a thing, at least, is possible in physics.

If a vessel is filled to its brim with warm water, it would seem to be impossible to put anything more in it; but, three ounces of sugar may be gradually introduced, then two ounces of the *bitartrate of potash*, then nine drachms of *green vitriol*, six drachms of *nitre*, six drachms of *smelling-salts*, two drachms and a half of *alum*, and a drachm and a half of *borax;* and still the vessel will not run over: the *bulk* will be the same, but the weight will be greater.

There is also a moral fuller than full. A man has enough money for all moderate purposes, and he is comparatively happy; and anon, he sets about accumulating a great deal more, and makes himself miserable. To the glutton and the miser, therefore, we say: WHEN YOU HAVE ENOUGH, STOP!

